# Azolyacetones as Precursors to Indoles and Naphthofurans Facilitated by Microwave Irradiation with Simultaneous Cooling

**DOI:** 10.3390/molecules14082976

**Published:** 2009-08-11

**Authors:** Saleh Mohammed Al-Mousawi, Morsy Ahmed El-Apasery

**Affiliations:** Department of Chemistry, Faculty of Science; University of Kuwait, Safat, 13060, P.O. Box 12613, Kuwait

**Keywords:** microwave irradiation, aminoindole, naphthofuran, azolylacetone

## Abstract

Phthalimide reacted with phenacyl bromide under microwave irradiation to yield phenacyl isoindolidene-1,3-dione (**3b**), while **3a** reacted with phenylhydrazine to yield the phenylhydrazone **4** that was readily converted into indoylphthalimide **8**. Similarly *N*-benzotriazolylacetone (**6a**) reacted with phenyl hydrazine to yield the phenylhydrazone **7a** that was converted into indoylbenzotriazole **9**. Treatment of **8** with hydrazine hydrate afforded a mixture of phthalhydrazide **10** and 3-amino-2-methylindole (**11**). Reacting enaminone **13** with naphthoquinone (**14**) afforded the aryl naphthofuran **17**. The possibility of the formation of the aldehyde **18** was excluded based on HMQC, which revealed that the carbonyl carbon is not linked to any hydrogen.

## Introduction

Azolylketones are versatile reagents and their chemistry has received considerable attention [1,2,3,4]. In previous work from our laboratories [5,6,7,8,9], we have investigated possible utility of 2-(2-oxopropyl)isoindole-1,3-dione (**3a**) and 1-benzotriazol-1-yl-propan-2-one (**6a**) for synthesis of polyfunctional heteroaromatics. In conjunction of this work we report here on utility of compounds **3** and **6** as precursors to substituted indoles and naphthofurans. However, we found that the reactions of azolylketones were rather sluggish at room temperature, affording the final naphthofurans in moderate yields. During the course of our explorations, we observed that the rate and the yield of these reactions could be greatly improved when performed under microwave irradiation in combination with simultaneous cooling at 30 °C. In this contribution, we would like to disclose the preliminary results of our investigations.

## Results and Discussion

**Scheme 1:**
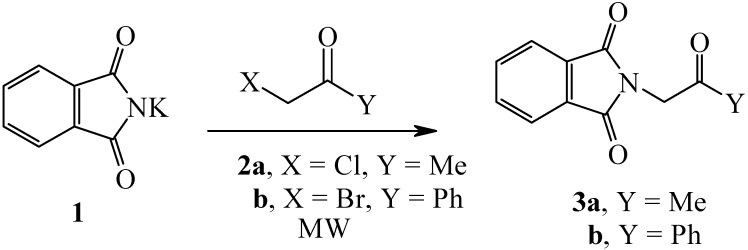
Preparation of 2-(2-Oxo-2-phenylethyl)isoindole-1,3-dione.

Reacting potassium phthalimide (**1**) with phenacyl bromide (**2b**) either by refluxing in dimethylformamide (DMF) for 30 minutes or in microwave oven (MW) at 55 °C for 10 minutes afforded the phenacyl isoindolidinedione (**3b**) (*cf*. Scheme 1).

**Scheme 2:**
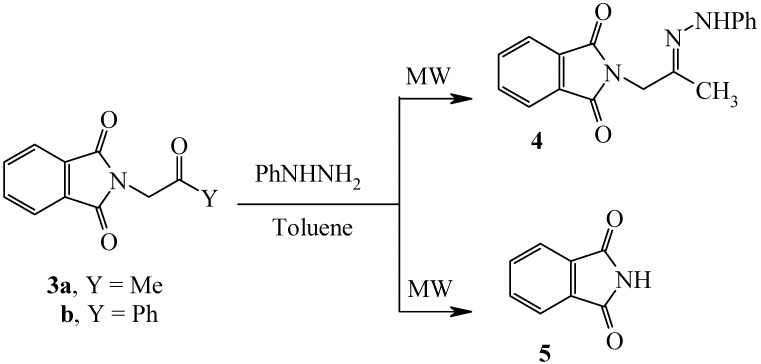
Preparation of 2-[2-(Phenylhydrazono)propyl]isoindole-1,3-dione.

Compound **3a** [6,7] reacted with phenylhydrazine under MW at 130 °C for 20 minutes to yield the phenylhydrazone **4** [7] in 74% yield, while similar treatment of **3b** [10,11,12] resulted in formation of phthalimide (**5**) (*cf.* Scheme 2). 

**Scheme 3:**
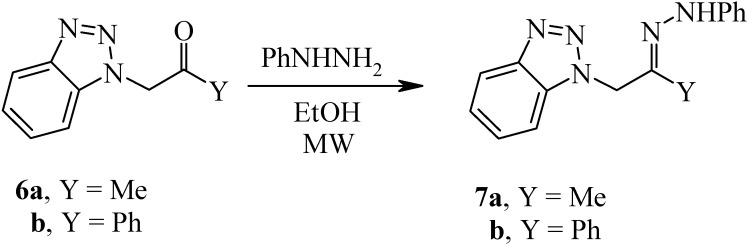
Preparation of *N*-(2-Benzotriazol-1-yl-1-methyl-ethylidene)-*N'*-phenylhydrazine.

Similarly, *N*-benzotriazolylacetone (**6a**) reacted with phenylhydrazine under MW at 130 °C for 20 minutes to yield the phenylhydrazone **7a** in 89% yield (*cf.* Scheme 3).

**Scheme 4:**
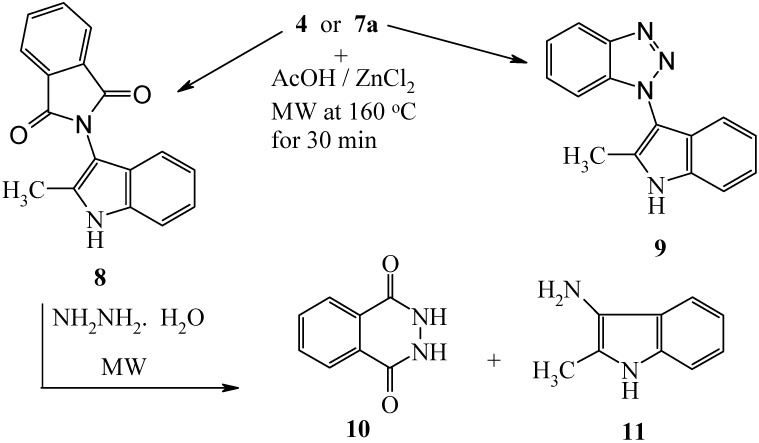
Synthesis of 1-(2-Methyl-1*H*-indol-3-yl)-1*H*-benzotriazole.

Heating of **4** or **7a** with zinc chloride and a few drops of acetic acid under microwave irradiation at 160 °C for 30 minutes afforded compounds **8** or **9** in 72% and 88% yields, respectively. Despite Katritzky’s reports [13,14] that benzotriazole substituents are masked halides, we failed to replace the benzotriazolyl moiety in the formed indole under a variety of conditions. It is of value to report that compound **8** has been previously obtained via pyrolysis of 4,5-disubstituted 1-phthalimido-1,2,3-triazoles [15]. Treatment of **8** with hydrazine hydrate under MW at 130 °C for 20 minutes afforded phthalhydrazide (**10**) in 85% yield, along with a product which was identified as 3-amino-2-methylindole (**11**). This amine **11** has been previously prepared in two steps via nitration of 2-methylindole and subsequent reduction [16] (*cf.* Scheme 4).

**Scheme 5:**
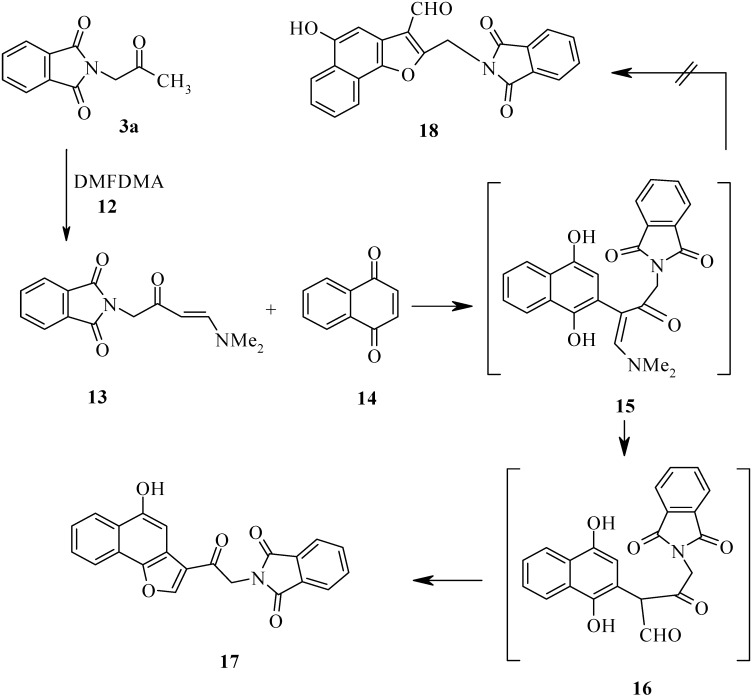
Synthesis of 2-[2-(5-Hydroxynaphtho[1,2-b]furan-3-yl)-2-oxoethyl]isoindole-1,3-dione.

In previous work we have shown that the methylene group in *N*-alkylazolylketones is activated toward electrophiles and that this activity is enhanced by microwave heating [17]. In continuation of this work we report on the synthesis of 2-(4-dimethylamino-2-oxo-3-butenyl)isoindole-1,3-dione as precursor to the title compounds. Thus, condensation of **3a** with dimethylformamide dimethyl acetal (DMF-DMA, **12**) has afforded enaminone **13** in 76% and 77% yields, either by refluxing in xylene for 8 h or by heating under MW without solvent at 180 °C for 20 min, respectively. We decided to investigate the effect of microwave irradiation, keeping the temperature at 30 °C. This should also allow us to maintain the maximum power input of 70 W during the full run of the irradiation. To ensure a correct temperature measurement, a fiber optic sensor was used. Using the same reagent ratios and solvent as these for the reaction run at room temperature, we performed the reaction of enaminone **13** with naphthoquinone (**14**) under microwave irradiation with simultaneous cooling to 30 °C. The reaction proceeded smoothly, furnishing the corresponding arylnaphthofuran product **17** in increased yield (66%); this is 52% higher compared to the conventional reaction at room temperature. It is noteworthy that the reactions could be performed using the irradiation power of 70 W continuously during the whole run, as the high power level was needed to maintain the temperature at 30 °C because of the efficient external cooling. The differences in yield could be attributed to a lower rate of decomposition of the compounds when using simultaneous cooling. The possibility of the formation of the aldehyde **18** was excluded based on HMQC, which revealed that the carbonyl carbon is not linked to any hydrogen (*cf.* Scheme 5). 

## Conclusions

In summary, we could extend Fisher’s indole reaction to enable the synthesis of 2-(2-methyl-1H-indol-3-yl)isoindole-1,3-dione and 1-(2-methyl-1H-indol-3-yl)-1H-benzotriazole using microwave irradiation. It is worthwhile to mention here that thermal and microwave assisted reactions were conducted in different solvents, with the exception of **13**, which was prepared by microwave heating without solvent. We have also revealed that the reactions described have proceeded to completion in a much shorter time when irradiated in a focused microwave oven. Moreover, the microwave assisted reactions produced somewhat higher yields than those obtained by conventional heating.

## Experimental

### General

Melting points are uncorrected. All the reactions were conducted under microwave irradiation in heavy-walled Pyrex tubes (capacity 10 mL) fitted with PCS caps. Microwave heating was carried out with a single mode cavity Explorer Microwave Synthesizer (CEM Corporation, NC, USA), producing continuous irradiation and equipped with simultaneous external air-cooling system. Compound **17** was prepared in a microwave irradiation experiment which was carried out in a dedicated CEM-Discover-Coolmate monomode microwave apparatus operating at a frequency of 2.45 GHz with continuous irradiation power from 0 to 300 W. The reaction was carried out in an open 10 mL double walled glass vial, which was cooled to 10 °C using a microwave transparent cooling liquid. The temperature was measured with a fiber-optic device inserted into the reaction vessel. IR spectra were recorded in KBr disks using a Perkin-Elmer System 2000 FT-IR spectrophotometer. ^1^H-NMR (400 MHz) and ^13^C-NMR (100 MHz) spectra were recorded on a Bruker DPX 400, super-conducting NMR spectrometer in CDCl_3_ or DMSO as solvent and TMS as internal standard; chemical shifts were reported in d units (ppm). Mass spectra were measured on a VG Autospec-Q (high resolution, high performance, tri-sector GC/MS/MS). Microanalyses were performed on a LECO CHNS-932 Elemental Analyzer. Compounds **3a**, **6a,b** and **7b** have been reported earlier and proven to be identical with the products obtained here [1,6,7,13,18]. 

### 2-(2-Oxo-2-phenylethyl)isoindole-1,3-dione *(**3b**)*

*Thermal method*: A mixture of phthalimide potassium salt (1.85 g, 0.01 mol) and phenacyl bromide (1.99 g, 0.01 mol) in dry DMF (20 mL) was mixed gently until the exothermic reaction ceased. The mixture was heated for 30 min, filtered and washed with water. The solid product was collected and crystallized from EtOH to give a yield of 95%, mp 160-164 °C (all data agreed with the published values [11,12]).

*Microwave method*: A mixture of phthalimide potassium salt (1.85 g, 0.01 mol) and phenacyl bromide (1.99 g, 0.01 mol) in dry DMF (2 mL) was irradiated in focused microwave at 55 °C for 10 min. The build-up of pressure in the closed reaction vessel was carefully monitored. After the irradiation, the reaction tube was cooled with high-pressure air through a built-in system in the instrument until the temperature had fallen below 50 °C. The crude product was poured onto water and the solid product, so formed, was collected by filtration and crystallized from EtOH to give a 68% yield of the target compound.

### General method for preparation of compounds ***4*** and ***7a***

A dried heavy-walled Pyrex tube containing a small stir bar was charged with compounds **3a** or **6a** (0.01 mol), phenylhydrazine (1.08 g, 0.01 mol)) and ethanol (2 mL) or toluene (2 mL). The tube containing the reaction mixture was fitted with a PCS cap and then it was exposed to microwave irradiation at 130 °C for 20 min. The solvent was removed and the residue, when cooled, deposited a solid, which was crystallized from ethanol.

*2-[2-(Phenylhydrazono)propyl]isoindole-1,3-dione* (**4**): mp 158-160 °C; IR (KBr, cm^-1^): 3321 (NH), 1774 (CO); MS (EI) m/z: (%) 293 (M+, 100%);^1^H-NMR (DMSO-*d_6_*): δ = 1.90 (s, 3H, CH_3_), 4.37 (s, 2H, CH_2_), 6.85 (t, 1H, J = 7.2 Hz, phenyl-H), 6.72 (d, 2H, J = 7.8 Hz, phenyl-H), 6.94 (t, 2H, J = 7.8 Hz, phenyl-H), 7.87-7.88 (m, 2H, phthalmidyl-H), 7.92-7.93 (m, 2H, phthalmidyl-H) 8.90 (s, 1H, NH), Anal. Calcd. for C_17_H_15_N_3_O_2_ (293.32): C, 69.61; H, 5.15; N, 14.33. Found: C, 69.24; H, 5.36; N, 13.94.

*N-(2-Benzotriazol-1-yl-1-methyl-ethylidene)-N'-phenylhydrazine* (**7a**): mp 128-130 °C; IR (KBr, cm^-1^): 3359 (NH), LC-MS 265 (M+, 58%);^1^H-NMR (DMSO-*d_6_*): δ = 1.84 (s, 3H, CH_3_), 5.53 (s, 2H, CH_2_), 6.68 (t, 1H, J = 7.2 Hz, arom-H), 6.96 (d, 2H, J = 8.4 Hz, arom-H), 7.11 (t, 2H, J = 7.6 Hz, arom-H), 7.39 (t, 1H, J = 7.2 Hz, arom-H), 7.53 (t, 1H, J = 7.2 Hz, arom-H), 7.79 (d, 1H, J = 8.4 Hz, arom-H), 8.07 (d, 1H, J = 8.4 Hz, arom-H), 9.10 (s, 1H, NH), ^13^C-NMR (DMSO-*d_6_*): δ = 13.61 (CH_3_), 54.24 (CH_2_), 110.81, 112.42, 118.80, 119.18, 123.94, 127.34, 128.79, 133.23, 138.61, 145.32, 145.65.

### General method for preparation of compounds ***8*** and ***9***

A dried heavy-walled Pyrex tube containing a small stir bar was charged with compounds **4** or **7a** (0.01 mol), zinc chloride (1.36 g, 0.01 mol) and acetic acid (2 mL). The tube was exposed to MW at 160 °C for 30 min. The crude product was poured onto water, and the solid product, so formed, was collected by filtration and crystallized from toluene.

*2-(2-Methyl-1H-indol-3-yl)isoindole-1,3-dione* (**8**): mp 222-224 °C; IR (KBr, cm^-1^): 3278 (NH), MS (EI) m/z: (%) 276 (M+, 100%);^1^H-NMR (DMSO-*d_6_*): δ = 2.77 (s, 3H, CH_3_), 6.95 (t, 1H, J = 7.2 Hz, indolyl -H), 7.07 (t, 1H, J = 7.2 Hz, indolyl -H), 7.23 (d, 1H, J = 8.0 Hz, indolyl -H), 7.36 (d, 1H, J = 8.0 Hz, indolyl-H), 7.91-7.93 (m, 2H, phthalmidyl-H), 7.98-8.00 (m, 2H, phthalmidyl-H) 11.41 (s, 1H, NH), ^13^C-NMR (DMSO-*d_6_*): δ = 11.07 (CH_3_), 103.74, 111.13, 117.09, 119.28, 120.93, 123.43, 124.75, 125.36, 128.24, 128.93, 131.85, 133.27, 133.95, 134.65, 163.49. Anal. Calcd. for C_17_H_12_N_2_O_2_ (276.29): C, 73.90; H, 4.38; N, 10.14. Found: C, 73.83; H, 4.38; N, 10.05.

*1-(2-Methyl-1H-indol-3-yl)-1H-benzotriazole* (**9**): mp ›300 °C; IR (KBr, cm^-1^): 3280 (NH), MS (EI) m/z: (%) 248 (M+, 30%);^1^H-NMR (DMSO-*d_6_*): δ = 2.30 (s, 3H, CH_3_), 7.03 (t, 1H, J = 7.2 Hz, arom-H), 7.07 (d, 1H, J = 7.8 Hz, arom-H), 7.16 (t, 1H, J = 8.4 Hz, arom-H), 7.49-7.75 (m, 4H, arom-H), 7.56 (t, 1H, J = 7.8 Hz, arom-H), 8.16 (d, 1H, J = 8.4 Hz, arom-H), 11.75 (s, 1H, NH), ^13^C- NMR (DMSO-*d_6_*: δ = 11.33 (CH_3_), 109.38, 111.05, 112.10, 116.33, 119.21, 120.02, 120.67, 122.20, 124.70, 128.20. 131.65, 134.10, 134.51, 145.49.

### 2-Methyl-1H-indol-3-ylamine *(11)*

A mixture of compound **8** (2.76 g, 0.01 mol) and hydrazine hydrate (1.43 g, 0.02 mol) in EtOH (3 mL) was irradiated by focused microwave at 130 °C for 20 min. The solid product, so formed, was collected by filtration to separate the side product [phthalhydrazide (**10**)]. The filtrate was evaporated under vacuum to remove the solvent and the product **11** was isolated using column chromatography (EtOAc – CHCl_2_) (all data were in agreement agreed with the published values [16]).

### 2-(4-Dimethylamino-2-oxobut-3-enyl)isoindole-1,3-dione *(**13**)*

*Thermal method*: A mixture of phthalimidoacetone (2.03 g, 0.01 mol) and DMF-DMA (1.19 g, 0.01 mol) in xylene (5 mL) was heated under reflux for 8 h. The solid product was collected by filtration and crystallized from EtOH. The reaction gave yellow crystals, yield (76%), mp 159-162 °C (literature mp 155 °C [18]); IR (KBr) ν 1769 (CO), 1714 (CO), 1660 (CO) (cm^-1^); ^1^H-NMR (DMSO-*d_6_*): δ (ppm) 2.72 (s, 3H, CH_3_), 3.05 (s, 3H, CH_3_), 4.40 (s, 2H, CH_2_), 5.04 (d,1H, *J = 12* Hz, CH), 7.61 (d,1H, *J = 12* Hz, CH), 7.85-7.91 (m, 4H, phthalimidyl-H); MS (EI) *m*/z (%): 258.1[M^+^, 20%], 160(20), 76(10); Anal. Calcd.*.* for C_14_H_14_N_2_O_3_: C, 65.11; H, 5.46; N, 10.85; Found: C, 65.18; H, 5.44; N, 10.76.

*Microwave method*: A mixture of phthalimidoacetone (2.03 g, 0.01 mol) and DMF-DMA (1.19 g, 0.01 mol) was irradiated by focused microwave at 180 °C for 20 min. The build-up of pressure in the closed reaction vessel was carefully monitored. After the irradiation, the reaction tube was cooled with high-pressure air through a built-in system in the instrument until the temperature had fallen below 50 °C. The solid product, so formed, was collected by filtration and crystallized from EtOH to give a 77% yield of the target compound.

### 2-[2-(5-Hydroxynaphtho[1,2-b]furan-3-yl)-2-oxoethyl]isoindole-1,3-dione *(**17**)*

*Thermal method*: A mixture of compound **13** (2.58g, 0.01 mol) and naphthoquinone (1.54g, 0.01 mol) was dissolved in glacial acetic acid (10 mL), then stirred over night at room temperature. The so formed crystals were collected by filtration and crystallized from dioxane. This compound was obtained in yield (52 %). mp 301-303 °C; IR (KBr)(cm^-1^): 3309 (OH), 1776 (CO), 1722 (CO), 1666 (CO); ^1^H-NMR (DMSO-*d_6_*): d 5.17 (s, 2H, CH_2_), 7.45 (s, 1H, H-4), 7.58 (t, 1H, *J=8.4Hz*, H-7), 7.70 (t, 1H, *J=8.4Hz*, H-8), 7.91-7.92 (m, 1H, H-14), 7.97-7.98 (m, 1H, H-13), 8.23 (d, 1H, *J=8.4Hz*, H-9), 8.25 (d, 1H, *J=8.4Hz*, H-6), 9.31 (s, 1H, H-2), 10.34 (s, 1H, OH); ^13^C-NMR (DMSO-*d_6_*): d 188.71 (C-10), 167.99 (C-12), 153.74 (C-2), 151.65 (C-5), 145.02 (C-9b), 135.28 (C-14), 132.02 (C-12a), 128.01 (C-8), 125.61 (C-7), 123.97 (C-5a), 123.89 (C-13), 123.80 (C-6), 121.19 (C-9a), 120.28 (C-3a), 120.08 (C-3), 119.85 (C-9), 100.12 (C-4), 45.23 (CH_2_, C-11); MS (EI) m/z (%) = 371 [M^+^]; Anal. Calcd. for C_22_H_13_NO_5_ (371.35); C, 71.16; H, 3.53; N, 3.77. Found C, 71.14; H, 3.65; N, 3.95.

*Microwave method*: A mixture of compound **13** (2.58 g, 0.01 mol), naphthoquinone (1.54 g, 0.01 mol), and glacial acetic acid (1 mL) was irradiated at 30 °C for 5 min, continuously at the maximum power of 70 W. After completion of the reaction the solvent was evaporated. The crude product was then collected by filtration and crystallized from dioxane to give a 66% yield of **17**. 

## Supplementary Files

Supplementary File 1
