# Al-Mousawi, S.M., *et al*. Azolylacetones as Precursors to Indoles and Naphthofurans Facilitated by Microwave Irradiation with Simultaneous Cooling. *Molecules* 2009, *14*, 2976-2984

**DOI:** 10.3390/molecules15010093

**Published:** 2009-12-28

**Authors:** Saleh Mohammed Al-Mousawi, Morsy Ahmed El-Apasery

**Affiliations:** Department of Chemistry, Faculty of Science; University of Kuwait, Safat, 13060, P.O. Box 12613, Kuwait

We realized that the title was incorrectly listed in our paper published in Molecules recently [1]. The correct title is indicated below:

**Azolylacetones as Precursors to Indoles and Naphthofurans Facilitated by Microwave Irradiation with Simultaneous Cooling**
