# Supplementary material for: Azolyacetones as Precursors to Indoles and Naphthofurans Facilitated by Microwave Irradiation with Simultaneous Cooling
Source: Molecules. 2009 Aug 11;14(8):2976–84. doi: 10.3390/molecules14082976 (PMC6255476; doi:10.3390/molecules14082976)
Supplement: Supplementary File 1 [file molecules-14-02976-s001.pdf]

Molecules **2010**, *15*, 93; doi:10.3390/molecules15010093

OPEN ACCESS

molecules

ISSN 1420-3049

www.mdpi.com/journal/molecules

Correction

Al-Mousawi, S.M., *et al*. Azolylacetones as Precursors to Indoles and Naphthofurans Facilitated by Microwave Irradiation with Simultaneous Cooling. *Molecules* 2009, *14*, 2976-2984

Saleh Mohammed Al-Mousawi * and Morsy Ahmed El-Apasery

Department of Chemistry, Faculty of Science; University of Kuwait, Safat, 13060, P.O. Box 12613, Kuwait

* Author to whom correspondence should be addressed; E-Mail: saleh.almousawi@yahoo.com; Tel.: +965-24985547; Fax: +965-24816482.

Received: 28 December 2009 / Published: 28 December 2009

We realized that the title was incorrectly listed in our paper published in Molecules recently [1]. The correct title is indicated below:

Azolylacetones as Precursors to Indoles and Naphthofurans Facilitated by Microwave Irradiation with Simultaneous Cooling

Reference

1. Al-Mousawi, S.M.; El-Apasery, M.A. Azolyacetones as precursors to indoles and naphthofurans facilitated by microwave irradiation with simultaneous cooling. *Molecules* **2009**, *14*, 2976-2984.

© 2010 by the authors; licensee Molecular Diversity Preservation International, Basel, Switzerland. This article is an open-access article distributed under the terms and conditions of the Creative Commons Attribution license (http://creativecommons.org/licenses/by/3.0/).
